# Tolerance to decentration of biaspheric intraocular lenses with refractive phase-ring extended depth of focus and diffractive trifocal designs

**DOI:** 10.1007/s00417-024-06458-1

**Published:** 2024-03-25

**Authors:** Alejandro Cerviño, Jose Juan Esteve-Taboada, Yi-Feng Chiu, Chuan-Hui Yang, Wen-Chu Tseng, William Lee

**Affiliations:** 1 Department of Optics & Optometry & Vision Sciences, University of Valencia, C / Dr. Moliner, 50., 46100 Burjassot, Valencia, Spain; 2 ICARES Medicus, Inc, Hsinchu County, Taiwan; 3 AST Products, Inc, Billerica, MA USA

**Keywords:** EDOF IOL, Trifocal IOL, Optical bench, In vitro, Optical quality, Presbyopia correcting IOL

## Abstract

**Purpose:**

This study aimed to investigate the in vitro tolerance to decentration of biaspheric intraocular lens (IOLs) with refractive phase-ring extended depth-of-focus (EDOF) and diffractive trifocal designs.

**Methods:**

This experimental study was carried out at the Department of Optics and Optometry and Vision Science, University of Valencia, Spain. The modulation transfer function (MTF) of the ETLIO130C EDOF and the TFLIO130C trifocal IOLs (AST Products Inc., Billerica, MA, USA) were determined at different levels of decentration for a given wavelength and pupil diameter using the PMTF optical bench (Lambda-X Ophthalmics, Nivelles, Belgium). The modulation transfer function (MTF) curves, the through-focus MTF curves, and the Strehl ratios were measured at 3-mm pupil aperture for 0.25-, 0.50- and 0.75-mm decentration.

**Results:**

The optical design of the trifocal TFLIO130C IOL is robust to small decentrations, with virtually no change in MTF response for 0.25 mm decentration. For greater decentration levels, the MTF response is slightly reduced with increasing decentration. The ETLIO130C EDOF design is robust to decentration, as the MTF response is only minimally affected when increasing the decentration up to 0.75 mm.

**Conclusions:**

MTF responses are slightly reduced with greater levels of decentration, but the range of focus provided by both trifocal and EDOF designs are preserved. The effects for average levels of decentration reported in the literature are minimum for both IOL designs.

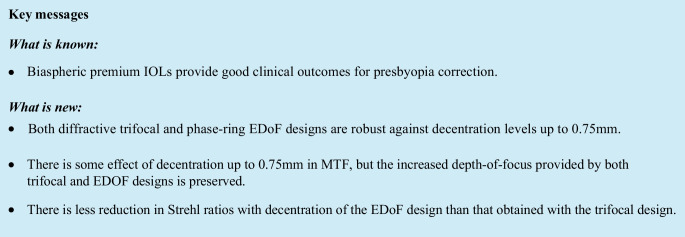



## Introduction

Enhanced depth-of-focus (EDOF) and trifocal intraocular lenses (IOLs) were introduced into the market to provide spectacle independence to cataract patients. Monofocal IOLs provide excellent visual acuity at one specific vergence distance but fail to provide good visual performance at all other vergences within the object space range. EDOF and trifocal IOLs improved the outcomes at these distances allowing an increased depth-of-field. Specifically, EDOF IOLs aimed to reduce dysphotopsia and avoid contrast sensitivity loss compared to trifocal models. A recent publication concluded that trifocal IOLs show better performance at near distances than hybrid multifocal-EDOF IOLs, but resulted in more photic disturbances [1]. Most multifocal and EDOF IOLs that were compared with a monofocal IOL show superior patient-reported spectacle independence, but patients also display decreased contrast sensitivity, reporting more visual phenomena, compared to those receiving monofocal IOLs [2].

Presbyopia-correcting IOLs are widely implanted following the demands in cataract surgery. Recent EDOF IOL models have been studied in vitro using optical metrics such as the modulation transfer function (MTF) or the point spread function (PSF) at different apertures [3–6]. One of the most important analyses that can be done using an optical bench is to analyze robustness to decentration to simulate common clinical situations when the lens is implanted into the eye. The analysis of the influence of decentration on the optical quality of Premium designs is crucial, since IOL decentration may be related to a reduction of overall postoperative optical quality and consequently on the visual performance of our patients [7–10].

The purpose of the present study was to analyze the effect of different levels of decentration in two new Premium biaspheric IOL designs, a refractive EDOF, and a diffractive trifocal, by means of through-focus MTF and Strehl ratios.

## Methods

### PMTF optical device

All the measurements were obtained using the PMTF optical device (Lambda-X Ophthalmics, Nivelles, Belgium). This instrument was designed to offer real-time and high-quality measurements of the MTF of IOLs following the guidelines specified in the International Organization for Standardization (ISO) standard normative [11]. It can also obtain the dioptric power of the IOL by measuring the magnification of the image of a known target, a procedure also suggested in the ISO standard.

**Fig. 1:**
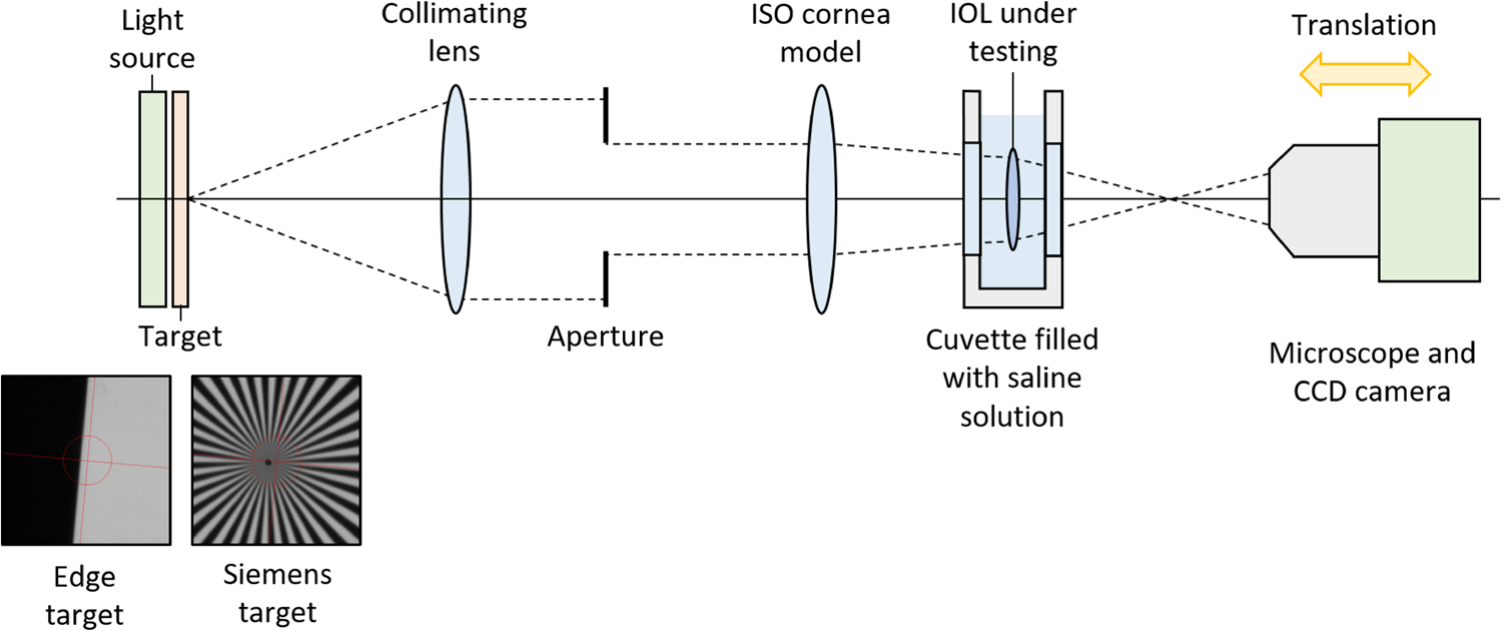
Schematic diagram of the PMTF optical device

Figure 1 shows a schematic diagram of the PMTF optical device. The light source was a LED based light source, with a wavelength of 550 nm (green color), and with a full width at half maximum (FWHM) of 10 nm. The target is the pattern that is imaged on the CCD camera through the ISO eye model. Two different targets were used: edge target to measure the MTF (as described below), and Siemens resolution target for the initial alignment of the eye model with the optical axis of the PMTF device. The ISO eye model is defined by the combination, i.e., optical association, of the cornea model and the IOL that is being measured. Different cornea models can be used in the instrument when measuring the IOLs performance. The particular cornea model that was used, described in detail below, fulfill the requirements established in the ISO standard. The aperture is a hole limiting the pupil diameter in the IOL plane. All measurements were carried out for a 3.0-mm pupil size, since benefits of presbyopia-correcting IOLs are intended for photopic light conditions and, according to the existing literature, the average pupil size in patients 48 years of age and above is 2.58 mm [12].

For the measurement, the IOL is placed into an insert, which in turn is embedded into a cuvette filled with saline solution, in our case, physiological saline solution Vitulia 0.9% (Laboratorios ERN, S.A., Barcelona, Spain).

Once the IOL is embedded into the cuvette filled with saline solution, the cuvette is placed onto an XY translation table which contains the cornea model. This assembly, that makes up the eye model, is compatible with the design rules specified in the ISO standard:

- The converging beam of the cornea model, when exposing a circular area of 5.15 mm ± 0.10 mm at an axial location that is between 26 and 28 mm in front of the focal point of the model cornea itself, taking the refractive index of the image space to be 1.336, produces a wavefront that is characterized by a value for the Zernike coefficient C(4,0) to within ± 0.020 μm of the intended value.
- The IOL front surface is placed at the axial location specified in the previous point.
- The converging beam from the cornea model is stopped down to expose a central circular area of the IOL having a diameter appropriate for the test to a tolerance of ± 0.1 mm.
- The IOL is placed in a liquid medium contained between two flat windows (cuvette filled with saline solution).
- The difference in refractive index between the IOL and the liquid medium is within 0.005 units of that under in situ conditions.
- The image plane falls in air, beyond the last window of the cuvette.

This eye model uses a well characterized standard thick lens as cornea model, in order to provide a spherical aberration of 0.295 μm. The dimensions of this lens are the following: first surface radius 51.22 mm, second surface radius − 28.00 mm, thickness 6.90 ± 0.05 mm. A value of 0.280 μm for the Zernike coefficient C(4,0), first-order spherical aberration, is described by Wang et al. [13] for an average human eye with a 6-mm entrance pupil. This eye model is recommended in the ISO standard for MTF testing of IOLs that are intended to have a certain amount of spherical aberration.

Finally, the microscope lens, that is adapted to the CCD camera, focuses on the image plane of the eye model, and forms an image into the CCD sensor. The microscope and the camera are mounted on a translation stage in order to change the plane of focus. The minimum translation step is 2.5 μm, and the CCD sensor provides images of 1040 × 1040 pixels. The PMTF considers IOLs made of material with refractive index of 1.46, placed in saline solution with refractive index of 1.336.

### Measurement of the imaging quality of IOLs

Imaging quality of IOLs can be specified either as resolution efficiency or as the MTF value at a specified spatial frequency. The PMTF instrument is compliant with the requirements and methodology described in the ISO standard for the MTF measurement of IOLs using the eye model previously described. According to these requirements, the MTF measurement is done by imaging an edge target and processing the image. The sharpness of the edge image is the result of a high-quality eye model (cornea model + IOL). A vertical edge gives the tangential MTF and a horizontal edge gives the sagittal MTF. In any case, for a rotationally-symmetric IOL there is no need for measuring both sagittal and tangential MTFs, given that by definition, and considering that no reference is taken in the orientation of the IOL when it is placed into the insert and cuvette, both MTF functions will be interchangeable.

Following ISO recommendations, the edge target image is focused to obtain maximum MTF at 50 lp/mm with a 3.0- ± 0.1-mm aperture and a light source with a peak wavelength within ± 10 nm of 550 nm (green), after ensuring that the IOL is in the correct position and the eye model is properly aligned with the instrument optical axis.

MTF can be also obtained in different axial positions close to the focus plane. Thus, a plot of MTF values at a given spatial frequency, for instance 50 lp/mm, in different axial positions in front of and behind the best focus plane, the so called through-focus MTF curve, was also obtained. A set of measurements of MTF, through-focus MTF, and Strehl ratio (a measure of the quality of optical image formation) were performed following the guidelines recommended in the ISO standard, with different decentration values of the IOL from the PMTF optical axis and a human-like aberrated cornea model with regard to spherical aberration.

The measurement methodology was also evaluated prior to the study by assessing the PMTF repeatability on a set of IOLs, taking repeated measurements both with and without removing the IOL from the cuvette between measures. Also, the repeatability of the manufacturing process was assessed by measuring several IOLs of the same design, obtaining that the variability of the manufacturing process was lower than the variability of the repeated measurements. These assessments of the method justify the analysis of one specimen of each IOL design, having in mind that checking the insertion of the lens and its positioning with respect to the PMTF optical setup is crucial.

### Intraocular lenses

The Asqelio™ EDOF Toric and Asqelio™ Trifocal IOLs used and measured were manufactured by AST Products Inc. (Billerica, MA, USA) via proprietary optic design and lathe-cutting of the same hydrophobic, low water content material. Table 1 displays the design and physical characteristics of the two IOL models and their nominal powers used for the purpose of the present study.

**Table 1:** Design and physical characteristics of the intraocular lenses used and measured

Brand name	Asqelio™^*^ EDOF Toric	Asqelio™ Trifocal
Model	ETLIO130C (Clear)	TFLIO130C (Clear)
Material	Single piece hydrophobic acrylic with UV absorber	
Overall length	13.0 mm	
Optic diameter	6.0 mm	
Optic design	Bi-aspheric surface with anterior toric and posterior phase-ring** profile, 360° sharp edge optic and haptic	Bi-aspheric surface with a posterior diffractive-ring** profile, 360° sharp edge optic and haptic
Refractive index	1.5	
ABBE number	50	
Water content	< 0.5%	
SA (spherical aberration)	–0.27 μm @ 6 mm	
IOL power used for this study (SE diopters)	+ 18.0D	+ 20.0D
Cylinder power	0.0 D	N/A
Add power	Extended depth of focus (EDOF)	Intermediate + 2.2 D Near + 3.3 D
Diffractive steps	N/A	15 rings in center 4.5 mm zone
Light distribution	N/A	50% for distance, 24% for intermediate and 26% for near
UV cut-off at 10%	382 nm	

*Trademark of AST Products, Inc

**AST Products, Inc.’s proprietary and patent-pending technologies

### Measurements

All the measurements for these IOLs have been carried out under the following conditions:

1. After ensuring correct IOL position and eye model alignment with the optical axis of the instrument, MTF was measured at each best focus position for a 3-mm aperture.
2. IOL was then decentered 0.25, 0.50, and 0.75 mm from the optical axis, and the MTF was measured at each best focus position in the decentered lens.
3. Strehl ratio values were obtained as the ratio between the area-under-the-curve (AUC) of the MTF of the IOL measured and the AUC of the corresponding diffraction-limited MTF.
4. Through-focus MTF curves were determined at 50 cy/mm spatial frequency. Horizontal axis was measured in diopters relative to the far focus for the centered lens.
5. Best intermediate and near foci were determined from the local maxima in the through-focus curves.
6. Environmental conditions in the lab were monitored to ensure temperature variations within the range 20.0 ± 1.0 °C.

## Results

**Fig. 2:**
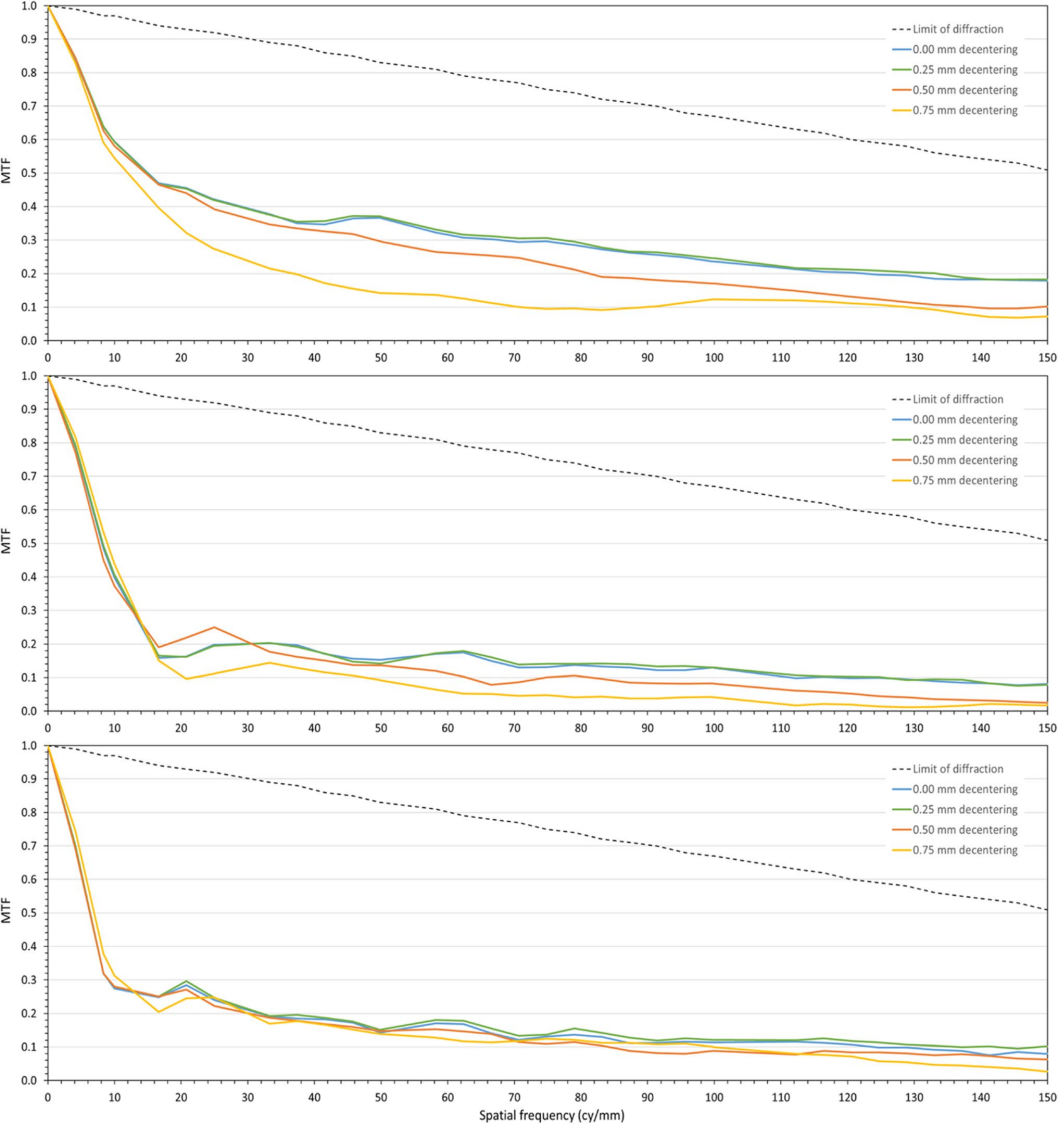
MTF measurements for the far (upper), intermediate (medium), and near (lower) foci of the trifocal IOL with the lens centered (blue line), and 0.25 (green line), 0.50 (orange line), and 0.75 mm (yellow line) decentration levels. Black dotted line represents the diffraction-limited response MTF

Figure 2 shows the MTF measurements obtained for the far (upper), intermediate (middle), and near (lower) foci of the trifocal IOL when the lens was centered (blue line), and also for three degrees of decentration from the optical axis of the PMTF: 0.25 (green line), 0.50 (orange line), and 0.75 mm (yellow line). Besides, MTF corresponding to the diffraction-limited response is shown as a black dotted line. The optical design of the lens is robust to small decentrations, as MTF response for 0.25 mm decentration is virtually the same as the one obtained in the centered case. For greater decentrations, MTF response is slightly reduced, being this reduction greater for increasing decentration levels, as expected. For instance, a decentration of 0.50 mm induces a 19% reduction in the MTF AUC corresponding to the far focus, while a 0.75-mm decentration implies a reduction of 44%. It is worth noting that MTF reduction for the near focus is lower than for the far focus. For instance, a decentration of 0.50 mm produces a reduction of only 13% in the MTF AUC corresponding to near, and just 22% for a decentration of 0.75 mm.

**Fig. 3:**
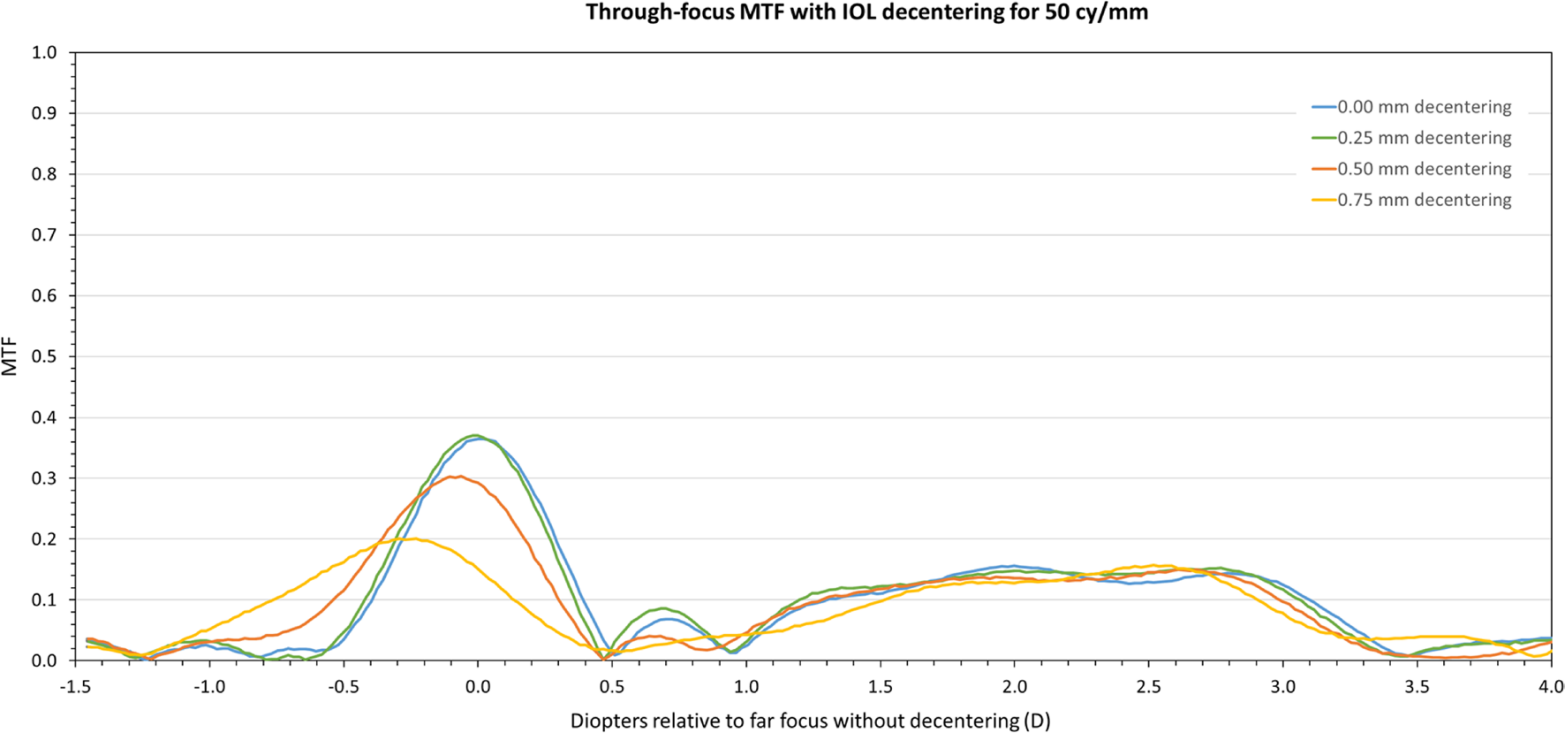
Through-focus MTF curve for a spatial frequency of 50 cy/mm for the trifocal IOL centered (blue line), and for 0.25 (green line), 0.50 (orange line), and 0.75 mm (yellow line) decentration levels

This behavior can also be seen in the through-focus MTF curve depicted in Fig. 3 for a spatial frequency of 50 lp/mm. As decentration increases MTF values do experience a reduction although the increased depth-of-focus provided by the trifocal lens is maintained throughout.

Table 2 shows the Strehl ratio values for both IOL designs studied, and for all foci and degrees of decentration. As was expected looking at the MTF curves, the Strehl ratio values for the trifocal IOL decrease as the decentration increases.

**Table 2:** Strehl ratio values for the trifocal and the EDOF lenses, and for different degrees of decentering with respect to the optical axis of the PMTF device. Best intermediate and near foci positions were determined from the local maxima of the through-focus curves

	Trifocal TFLIO130C focus			EDOF ETLIO130C focus		
Decentering (mm)	Far	Intermediate	Near	Far	Intermediate	Near
0.00	0.43	0.22	0.23	0.46	0.23	0.22
0.25	0.43	0.22	0.24	0.43	0.22	0.22
0.50	0.35	0.17	0.20	0.35	0.19	0.20
0.75	0.24	0.14	0.18	0.37	0.21	0.18

**Fig. 4:**
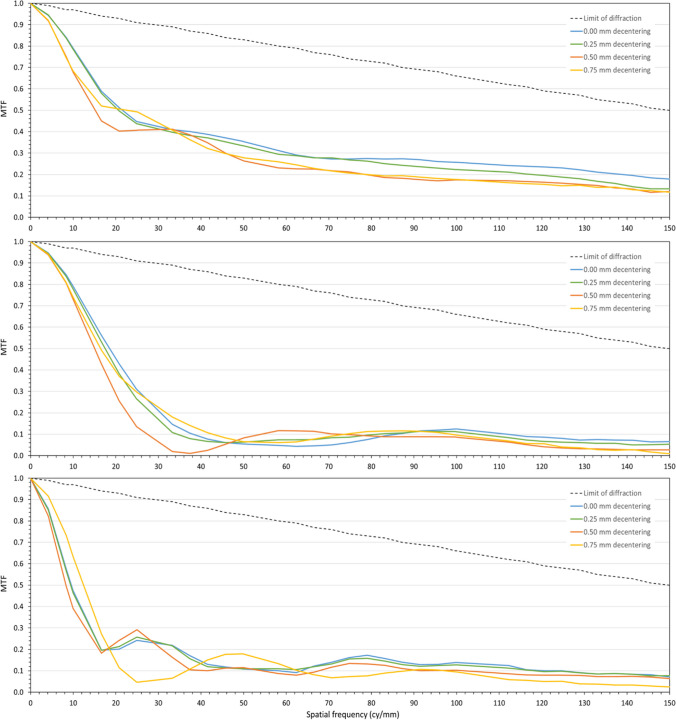
MTF measurements for the far (upper), intermediate (medium), and near (lower) foci of the EDoF IOL with the lens centered (blue line), and 0.25 (green line), 0.50 (orange line), and 0.75 mm (yellow line) decentration levels. Black dotted line represents the diffraction-limited response MTF

Figure 4 shows the MTF curves obtained for the far (upper), intermediate (middle), and near (lower) foci with the EDOF design. These figures follow the same color scheme as the previous ones, i.e., blue line for the centered case, and green, orange, and yellow lines for the lens decentered 0.25, 0.50, and 0.75 mm, respectively. Again, the diffraction limited MTF is shown for reference purposes with a black dotted line. The optical design of this EDOF IOL shows robustness to lens decentration, as the MTF response is only minimally affected when increasing decentration up to 0.75 mm. A decentration level of 0.50 mm, for instance, produces a reduction of 24% in the MTF AUC corresponding to the far focus, while a decentration of 0.75 mm produces a reduction of 20%. And with regard to the MTF AUC corresponding to the near focus, a decentration of 0.50 mm produces a reduction of 9%, and a decentration of 0.75 mm produces a reduction of 18%.

**Fig. 5:**
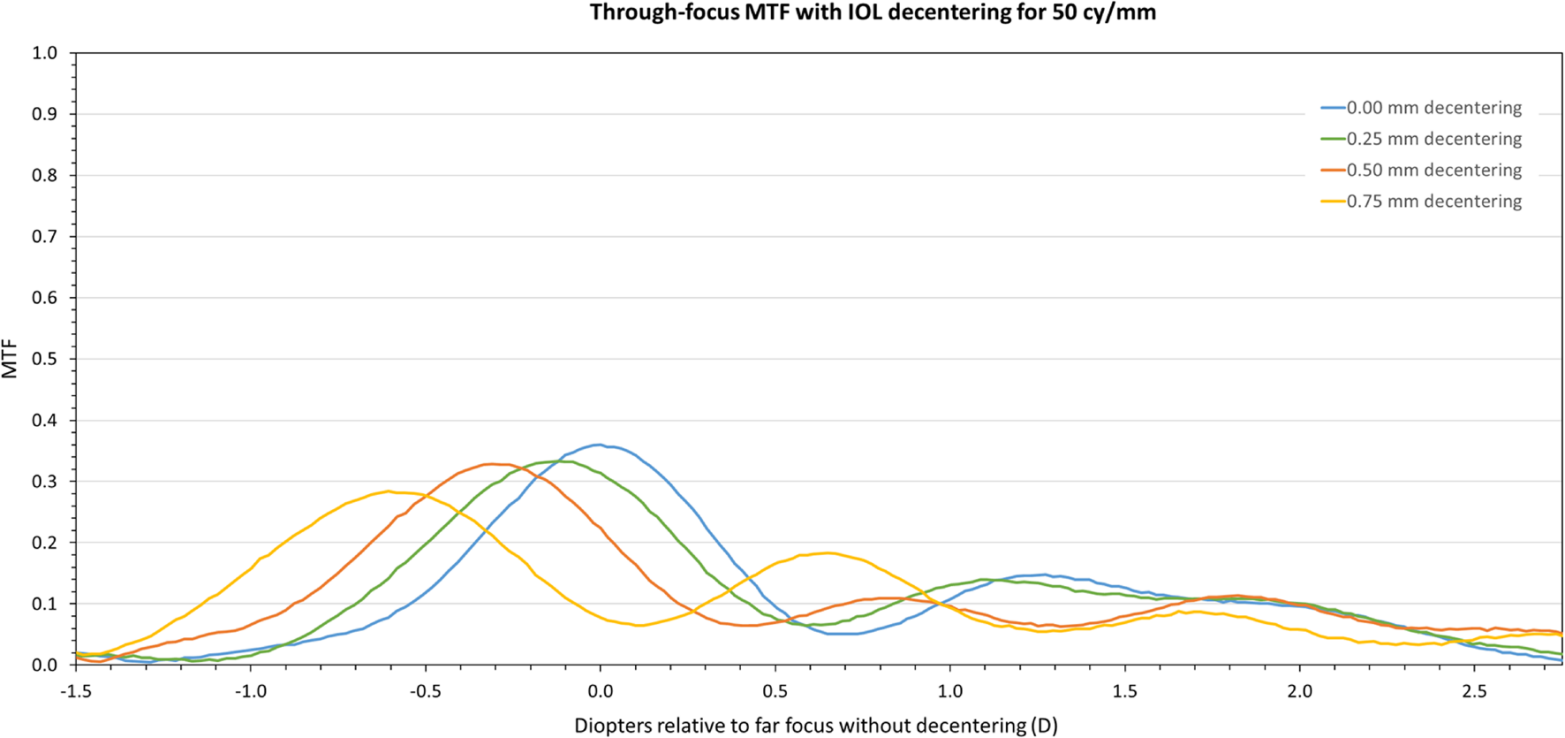
Through-focus MTF curve for a spatial frequency of 50 cy/mm for the EDoF IOL centered (blue line), and for 0.25 (green line), 0.50 (orange line), and 0.75 mm (yellow line) decentration levels

This performance can be also observed, as expected, in the through-focus MTF curve depicted in Fig. 5 for a spatial frequency of 50 lp/mm. As decentration increases to the measured limit of 0.75 mm, MTF values are slightly reduced, although the extended depth-of-focus behavior provided by the EDOF lens is maintained. In this sense, it is worth noting that although the EDOF design of this lens gives, by definition, a broader area with high MTF values (in the sense that no clear different foci are seen in the through-focus curve), best intermediate and near foci positions were determined from the local maxima of the through-focus MTF curve just for comparison purposes.

Table 2 (right side) shows the Strehl ratio values for the EDOF design, and for the different decentration values that were measured. As expected from MTF results, the reduction in the Strehl ratios observed with decentration is smaller than that obtained for the trifocal design.

## Discussion

Precise centration is essential to achieve optimal optical performance and improved visual outcomes with aspheric IOLs, since decentration of the aspheric IOLs tends to increase aberrations and worsen image quality [14, 15].

Xu et al. [16] reported mean decentration magnitudes of 0.27 ± 0.15 mm, while Crnej et al. found that three-piece IOLs are more likely to achieve greater decentration than one-piece IOLs [17].

On the other hand, the human eye is not a centered optical system, and it is recommended that cataract surgeons take this into account particularly when considering implantation of multifocal IOLs [18]. It was found that angle kappa greater than 0.5 mm in chord could significantly affect the quality of vision of patients implanted with premium IOLs [19]. In large population studies, average size of angle kappa ranges between 0.3 and 0.4 mm in chord [20–23], while angle alpha seems to be around 0.45 ± 0.21 mm in chord [23].

In the present study the effect of IOL decentrations up to 0.75 mm in the optical performance of two biaspheric Premium IOL designs, trifocal and EDOF, were analyzed in vitro for a given wavelength and pupil diameter.

Schmid et al. [6] analyzed the TECNIS Symfony (Johnson & Johnson Vision, Santa Ana, CA, USA), the AcrySof IQ Vivity (Alcon Labs, Fort Worth, TX, USA), and the LuxSmart Crystal (Bausch&Lomb, St. Louis, MO, USA) IOLs by means of through-frequency MTF, Strehl ratio, and US Air Force targets centered and with a decentration of 1 mm and a tilt of 5° as well as different apertures of 3 and 4.5 mm. Through-frequency MTF of Symfony was significantly better than the MTF curve of Vivity or LuxSmart, and this was true for misalignment, too, when IOLs were tilted to 5°. For a decentration of 1 mm, Symfony showed a dramatic decrease of MTF while Vivity and LuxSmart were less sensitive to decentration. However, the performance decentered was poor for all lenses.

Schmid et al. [5] assessed and compared the MTF of the AcrySof Vivity, LuxSmart Crystal, RayOne EMV (Rayner, UK), and Tecnis Eyhance (Johnson & Johnson Vision, Santa Ana, CA, USA) IOLs at 3- and 4.5-mm pupils. They concluded that Vivity and LuxSmart showed a larger depth-of-focus for their measuring conditions than Eyhance and RayOne EMV. The MTF peak was best for Eyhance and RayOne ERV with small apertures, while with larger apertures, RayOne EMV performed considerably worse. The same authors [10] analyzed the effect of decentration (1 mm) and tilt (5°), and found that RayOne EMV IOL was very robust against misalignment but had considerable deterioration of MTF for large apertures. Tilt and decentration decreased the performance of Eyhance significantly but had minor impact on the performance of Vivity and LuxSmart. At 4.5 mm of aperture, the MTF and the Strehl ratio decreased markedly for all IOLs compared to 3 mm aperture. The best MTF and Strehl ratio was obtained for the Eyhance IOL well centered for both sizes of aperture. They concluded that tilt and decentration had a major impact on the performance of Eyhance only, which performed best of all IOLs tested when well centered. They also indicated that with larger apertures, the performance of all IOLs decreased significantly.

Ortiz et al. [7] analyzed the effect of decentration (0.2 and 0.4mm) of a trifocal IOL, the AT LISA tri 839MP (Carl Zeiss Meditec AG, Jena, Germany), at 3 and 4.5 mm apertures. Their results demonstrated that the effect of decentration slightly reduced the optical quality of the lens when the decentration induced is less than 0.4 mm.

Tandogan et al. [8] also analyzed the same lens with different degrees of decentration (0.25, 0.50, 0.75, and 1 mm) at 3 and 4.5 mm apertures. They also found that for the trifocal lens, the optical quality at all distances was significantly reduced if decentration exceeded 0.75 mm, with intermediate focus showing the least reduction.

Ruiz-Alcocer et al. [9] assessed the effect of decentration and tilt combined with prior myopic ablations in the XACT Mono-EDOF IOL (Santen Pharmceutical Co Ltd) and the FineVision trifocal IOL (Physiol, Belgium) with the PMTF device. Specifically, 0.4 mm of decentration and 4° of tilt were measured. They reported that when decentration or tilt was induced, the trifocal IOL showed negligible changes but the EDOF IOL showed a − 0.50-D shift of the overall curve and concluded that the trifocal IOL was less affected by misalignments.

The present study is the first to provide in vitro optical performance outcomes of Asqelio™ biaspheric hydrophobic trifocal and EDOF designs IOLs. The results obtained show that the trifocal design is robust to small decentrations, with no change in MTF for 0.25 mm decentration, although for greater levels of decentrations, there is a slight reduction in MTF response while preserving the overall depth-of-focus behavior with the different decentration levels. Results also show a significant robustness to decentration of the EDOF design, with only small effects in MTF response when decentration is increased up to 0.75 mm. MTF values are slightly reduced as decentration increases, but the extended depth-of-focus behavior provided by the EDOF lens is maintained, showing less reduction in Strehl ratios with decentration than that obtained with the trifocal design.

In conclusion, for the IOLs analyzed, there is a slight effect in MTF with decentration, being greater as decentration level increases, but the increased depth-of-focus provided by both trifocal and EDOF designs is preserved. The effects for the average levels of IOL decentration following cataract surgery reported in the literature are minimum for both IOL designs.
